# Reliable energy and responsive built environment: the missing links in COVID-19 response in resource-limited settings

**DOI:** 10.1186/s41182-020-00255-2

**Published:** 2020-08-12

**Authors:** Arvind Vashishta Rinkoo, Dinesh Songara, Arnika Sharma, Biswa Ranjan Patnaik, Rajesh Ranjan Singh, Rakesh Kumar Srivastava

**Affiliations:** The Wadhwani Initiative for Sustainable Healthcare (WISH), Building No. 24 (3rd floor), Okhla Industrial Estate, Phase-III, New Delhi, 110020 India

**Keywords:** COVID-19, Energy insecurity, Built environment, Primary healthcare, Low- and middle-income countries

## Abstract

Irrespective of how the COVID-19 pandemic evolves over time across the globe, our past experiences with comparable zoonotic diseases demonstrate the significance of having resilient primary healthcare systems to successfully respond to public health emergencies. However, literature corroborates that in low- and middle-income countries, the primary healthcare systems are plagued with significant energy insecurity and inadequate built environment. These gaps in reliable energy and responsive built environment in primary healthcare systems are exacerbated during disruptive public health emergencies such as COVID-19. In this letter, we discuss the way forward to address these gaps and the policy and practical implications thereof.

## Introduction

Irrespective of how the COVID-19 pandemic evolves over time across the globe, our past experiences with comparable zoonotic diseases with epidemic potential demonstrate the significance of having resilient primary healthcare systems to successfully respond to public health emergencies [1]. During such crises, robust primary healthcare systems, more so in resource-limited settings, are prerequisites to not only put in place effective infection prevention and control (IPC) measures at the last-mile over a sustained period of time, but also provide appropriate care and treatment to patients with mild-to-moderate symptoms thus reducing the pressure on already overwhelmed secondary and tertiary care systems [2]. In this letter, based on the review of the available literature, authors attempt to establish reliable energy and responsive built environment as the two important missing links in the primary healthcare systems of the low- and middle-income countries (LMIC) of the world in the context of COVID-19 response and discuss the way forward to address these gaps and the policy and practical implications thereof.

The World Health Organization guidelines include availability of reliable energy and adequate built environment under core component 8 of the “minimum” requirements at primary care level for implementing “basic” IPC measures to prevent and reduce the transmission of infectious diseases that pose global threats [2]. However, literature corroborates that in LMIC, the primary healthcare systems are plagued with significant energy insecurity and inadequate built environment [3, 4]. In fact, the lack of electrification leaves many primary healthcare facilities in LMIC with inadequate power provision for even basic services [5, 6]. Likewise, assessment of infrastructure in public health facilities in LMIC reveals inadequate space and physical facilities for even essential service provisions with a large proportion of facilities requiring urgent major renovation or complete reconstruction [7, 8].

These gaps in primary healthcare systems are exacerbated during disruptive public health emergencies such as COVID-19 [9]. Isolation and therapeutic wards have been reactively setup in different parts of the world for isolating and treating asymptomatic and mild-to-moderate cases of COVID-19. In many situations, particularly in low-resource last-mile settings, non-health facilities such as schools, community centers, and government buildings have been “hurriedly” converted into alternate care sites (ACSs) to address the burgeoning COVID-19 crisis. During COVID-19 like public health calamities, developing ACS is a well-recognized necessary step to provide appropriate care [9]. However, basic planning and preparedness should be an integral part of such recourse. Employing purely a conceptual approach (such as “we can use this school or that community centre,” without any underlying thought process) can be a major stumbling block in successful establishment and commissioning of these ACSs [10]. In fact, should community transmission become widespread among the rural populations of the LMIC, such “reactive” approaches may prove inadequate or even counterproductive. Not originally designed to serve as isolation or therapeutic wards, these ACSs in low-resource settings generally lack basic sanitation and infection control facilities [2, 9]. Moreover, most of these sites lack adequate supply of reliable energy to perform even basic IPC practices [10]. In effect, due to unreliability of the conventional grid, these sites mostly rely on fuel-operated generators resulting in unsustainably high operational costs.

## How can we fill these gaps?

With the pandemic gaining momentum in LMIC, it is imperative to devise ways to urgently fill the existing gaps in energy and built environment in respect of COVID-19 response in low-resource settings. In this regard, innovation holds the key—we have to find new solutions for the old problems. Green public health infrastructure—building sustainable, impactful, and innovative solutions at the nexus of physical infrastructure, energy, and public health to leapfrog the existing gaps—that is customizable and cost-effective and can work at multiple scales may be the way forward [2, 3, 6, 9]. In resource-limited settings, the green public health infrastructure concept can be embedded into the COVID-19 response primarily in two ways.

First approach can be to undertake a rapid energy-infrastructure gap assessment of existing earmarked health or non-health facilities, as applicable, to handpick the smartest and most plausible solutions in terms of sustainable energy and building performance to improve the overall resiliency, efficiency, and effectiveness of these facilities as COVID-19 care sites. In context of sustainable energy, technological advances in decentralized solar energy offer a plethora of exciting cost-effective replicable solutions for last-mile facilities not served or underserved by the conventional grids [3, 6]. Likewise, infrastructure upgradation or expansion can be quickly undertaken to improve the overall performance of the earmarked facilities. The transformed facilities should have well-demarcated entry and exit zones, patient care areas, and sanitation and waste disposal facilities [2]. Moreover, the resulting built infrastructure should ensure climate responsiveness, optimal thermal comfort, energy efficiency, adequate ventilation, and compliance with IPC guidelines and spatial recommendations for setting up COVID-19 isolation or therapeutic wards, as applicable. As time is of essence in context of COVID-19 response, ease of setting up the infrastructure and integrating it into the existing facility has to be ensured. The other approach can be to start anew. Based on specific goals and local needs in respect of COVID-19 response, prefabricated portable infrastructure can be quickly and cheaply erected. Compliance with IPC guidelines and spatial recommendations should be ensured [2].

## Are we there?

Solution exists in the form of green health infrastructure, encompassing decentralized solar energy systems and improved building design and performance, to address the pressing issues of energy insecurity and inadequate built environment in the context of COVID-19 response at the last-mile public health settings in LMIC [2, 6, 9, 11]. However, having a solution is one thing, integrating it into the existing system and making it a win-win for all the stakeholders is a different ballgame. In fact, to reach a level of implementation maturity at which meaningful improvement in quality of COVID-19 response in low-resource settings can be expected, numerous challenges to adoption of these solutions in LMIC have to be proactively addressed [6]. Strong political will and policy reforms—at both the domestic as well as international levels—could play a catalytical role by addressing political, regulatory, and economic barriers to adoption of green public health infrastructure in resource-limited settings [12]. Stepped up financing from the public and private sectors alike and better in-country planning and collaboration and domestic leadership are required to shift scarce available resources into these innovative solutions and thus circumvent the technical, financial, and economic barriers to adoption of these solutions [13].

Also, for green health infrastructure solutions to be sustainable and to incrementally contribute to resiliency of primary healthcare systems not only to COVID-19 breakout but also to future public health calamities, public health leaders in LMIC need to realize that measures and solutions aimed at ensuring availability of reliable energy and responsive built environment in resource-limited settings should be an integral part of a continuous well-planned preemptive process rather than reactionary to a pandemic breakout (in this case, COVID-19) [9, 11]. Such an approach is desirable as solutions around green public health infrastructure have the potential to be game changers in the COVID-19 era and “beyond” for primary healthcare systems of LMIC, especially in the last-mile settings [1, 11]. In order to sustainably maintain or even enhance green public health infrastructure for ensuring access to responsive built environment and reliable energy aimed at bringing meaningful improvements in public health responses to COVID-19 like emergencies in LMIC, one of the plausible ways forward may be to explore new sustainable and cost-effective business models addressing local needs and contexts, preferably centered around public private partnerships [14]. Also, scaling up of local capacities in the context of green health infrastructure would be crucial if these solutions have to be financially sustained and used efficiently in resource-limited settings of the world. Local capacity building, to an extent, would also address the technical and geographical barriers to adoption of these solutions at the last-mile in LMIC.

To formalize these solutions, fraternities of architects, civil engineers, and renewable energy specialists should be an integral part of the decision-making process at appropriate levels concerning public health emergencies’ preparedness and response. Academia, nongovernment organizations, and government departments working in the realm of green public health infrastructure should be closely involved in policy formulation and implementation. Such representations ought to be ensured in all countries, especially in LMIC where energy poverty and built environment gaps in public health are most profound, at both national as well as sub-national levels. Unfortunately, an all-inclusive decision-making process in context of COVID-19 response is lacking globally, as of now [15]. This needs to be changed—multistakeholderism governance is the key if last-mile gaps in reliable energy and responsive built environment are to be proactively bridged to contribute towards sustainable, resilient, and robust primary healthcare systems.

## Data Availability

Not applicable.

## Abbreviations

ACSs: Alternate care sites
IPC: Infection prevention and control
LMIC: Low- and middle-income countries

## References

[1] Zumla A, Dar O, Kock R, Muturi M, Ntoumi F, Kaleebu P (2016). Taking forward a ‘One Health’ approach for turning the tide against the Middle East respiratory syndrome coronavirus and other zoonotic pathogens with epidemic potential. Int J Infect Dis.

[2] World Health Organization. Minimum requirements for infection prevention and control (IPC) programmes. 2019. http://www.who.int/infection-prevention/publications/core-components/en. Accessed 12 May 2020.

[3] World Health Organization. Access to modern energy services for health facilities in resource-constrained settings: a review of status, significance, challenges and measurement. 2015. http://apps.who.int/iris/bitstream/handle/10665/156847/9789241507646_eng.pdf?sequence = 1&isAllowed = y. Accessed 17 May 2020.

[4] Leslie HH, Spiegelman D, Zhou X, Kruk ME (2017). Service readiness of health facilities in Bangladesh, Haiti, Kenya, Malawi, Namibia, Nepal, Rwanda, Senegal, Uganda and the United Republic of Tanzania. Bull World Health Organ.

[5] Adair-Rohani H, Zukor K, Bonjour S, Wilburn S, Kuesel AC, Hebert R (2013). Limited electricity access in health facilities of sub-Saharan Africa: a systematic review of data on electricity access, sources, and reliability. Glob Health Sci Pract.

[6] Dholakia HH (2018). Solar powered healthcare in developing countries. Nat Energy.

[7] Yahya T, Mohamed M (2018). Raising a mirror to quality of care in Tanzania: the five-star assessment. Lancet Glob Health.

[8] Armenta B, Rathi N, Assasnik N, Kamimura A (2018). Structural quality of healthcare facilities in India. Int J Health Care Qual Assur.

[9] World Health Organization. Role of primary care in the COVID-19 response. 2020. http://iris.wpro.who.int/bitstream/handle/10665.1/14510/Primary-care-COVID-19-eng.pdf. Accessed 19 May 2020.

[10] Iserson KV (2020). Alternative care sites: an option in disasters. West J Emerg Med.

[11] Hopman J, Allegranzi B, Mehtar S (2020). Managing COVID-19 in low- and middle-income countries. JAMA..

[12] Pegels A, Vidican-Auktor G, Lutkenhorst W, Altenburg T (2018). Politics of green energy policy. J Environ Dev.

[13] Schwerhoff G, Sy M (2017). Financing renewable energy in Africa-key challenge of the sustainable development goals. Renew Sust Energ Rev.

[14] National Academies of Sciences, Engineering, and Medicine. Engaging the private sector and developing partnerships to advance health and the sustainable development goals: proceedings of a workshop series. Washington, DC: The National Academies Press. 2017. http://www.ncbi.nlm.nih.gov/books/NBK464290/pdf/Bookshelf_NBK464290.pdf. Accessed 25 June 2020. 29116725

[15] Rajan D, Koch K, Rohrer K, Bajnoczki C, Socha A, Voss M (2020). Governance of the Covid-19 response: a call for more inclusive and transparent decision-making. BMJ Glob Health.

